# Idiopathic mesenteric phlebosclerosis associated with use of Chinese patent medicine: a case report and literature review

**DOI:** 10.3389/fmed.2025.1583341

**Published:** 2025-07-09

**Authors:** Li Lv, Ruo-yu Gao, Xi-qiu Yu

**Affiliations:** Department of Gastroenterology, Shenzhen Luohu People’s Hospital, Shenzhen, China

**Keywords:** idiopathic mesenteric phlebosclerotic colitis, Chinese patent medicine, colonoscopy, case report, colon

## Abstract

Idiopathic Mesenteric Phlebosclerosis Colitis (IMP) is indeed a rare and potentially life-threatening condition characterized by chronic ischemic changes in the colon, primarily due to calcification of the mesenteric veins. This disease is often associated with long-term use of certain herbal medicines, particularly in Asian populations, where its prevalence is notably higher. This report describes a 55-year-old male patient who has been taking Chinese patent medicine orally for an extended period to treat prostatitis. His primary symptom is diarrhea. Imaging studies revealed multiple linear calcifications in the mesenteric veins surrounding the ascending and transverse colon. Colonoscopic findings showed bluish-purple discolored mucosa and multiple ulcers of the colonic and rectal mucosa. Pathological biopsy indicated fibrous tissue proliferation in the mucosal lamina propria and thickening of some blood vessel walls accompanied by hyaline degeneration. Based on clinical presentation, CT, colonoscopy, and histopathological findings, the final diagnosis was confirmed as IMP.

## Introduction

Idiopathic Mesenteric Phlebosclerosis Colitis (IMP) is a rare form of ischemic colitis that primarily impacts the right hemicolon, characterized by the thickening of the colonic wall and calcification of the mesenteric veins. This condition is particularly prevalent in East Asia (1, 2), where there is a notable association with the long-term use of traditional herbal medicines, especially those containing geniposide, which may contribute to the disease’s pathogenesis (3, 4). Guo et al. (1) suggested that the use of herbal medicine might be linked to the development of IMP. A 2021 retrospective review of 240 individuals, predominantly of East Asian descent, conducted by Wang et al. (5) found that 78.7% of those diagnosed with IMP had used herbal medicines. The ileocecum and ascending colon are primary sites for absorbing water, nutrients, and toxins, which may explain why IMP typically affects the right colon and can extend to the descending colon, sigmoid colon, or rectum (6).

The clinical manifestations of IMP often include abdominal pain, diarrhea, and weight loss, which can lead to misdiagnosis as inflammatory bowel disease or other gastrointestinal disorders (7, 8). Diagnosis of IMP typically relies on imaging techniques such as computed tomography (CT) and colonoscopy. CT scans reveal characteristic features such as tree-like mesenteric venous calcifications and colonic wall thickening, while colonoscopy may show a dark purple discoloration of the mucosa along with erosions and ulcerations (6, 9). The histopathological examination often reveals fibrosis and calcification in the veins, which are critical for confirming the diagnosis (10, 11).

In this paper, we describe a case of IMP involving the entire colorectum, attributed to the prolonged oral intake of Chinese patent medicine. Although the disease was not identified in its early stages, several characteristic features of its progression are highlighted.

Treatment options for IMP remain controversial and vary based on the severity of the disease. While conservative management is often sufficient for asymptomatic patients or those with mild symptoms, surgical intervention, such as colectomy, may be necessary for patients presenting with severe symptoms or complications like intestinal obstruction (12, 13). A systematic review of cases indicates that a significant proportion of patients experience good recovery following conservative treatment, although some may require surgical intervention due to the severity of their condition (5, 14). In summary, idiopathic mesenteric phlebosclerosis is a complex condition that necessitates a high index of suspicion, particularly in patients with a history of herbal medicine use. Early diagnosis through appropriate imaging and histopathological evaluation is crucial for effective management and improved patient outcomes (15, 16).

## Case report

A 55-year-old male visited the Department of Gastroenterology at our hospital with a 2-year history of diarrhea. More than 2 years prior, the patient had diarrhea without obvious cause and passed yellow watery stools 6–8 times a day, more than 10 times a day. The patient’s medical history includes chronic prostatitis for over 10 years, for which he had been taking “Shubitong Capsules, Longjintonglin Capsules, Prostatitis Relief Capsules, and Qinglin Granules” as traditional Chinese medicine. He also had a 10-year history of hypertension, treated with oral metoprolol succinate. No obvious abnormality was found by physical examination.

Laboratory tests indicated hypokalemia (K 3.4 mmol/L), hyponatremia (NA 136 mmol/L), hypoalbuminemia (ALB 38.9 g/L), and positive fecal occult blood test (OB). The T cells spot test (T-Spot TB) was positive, while EB virus antibodies (anti-EB-IgM) and cytomegalovirus antibodies (anti-CMV-IgG and anti-CMV-IgM) were all negative. No significant abnormalities were found in erythrocyte sedimentation rate, C-reactive protein, complete blood count, coagulation profile, liver and kidney function tests, D-dimer, urinalysis, immunoglobulins, Immunoglobulin G4 (IgG4), gastrointestinal tumor markers, fasting blood glucose, stool bacterial cultures, anti-nuclear antibodies, antibody spectrum, anticardiolipin antibodies (ACA), β2-glycoprotein 1, antineutrophil cytoplasmic antibody (ANCA), and lupus anticoagulative substance.

**Figure 1:**
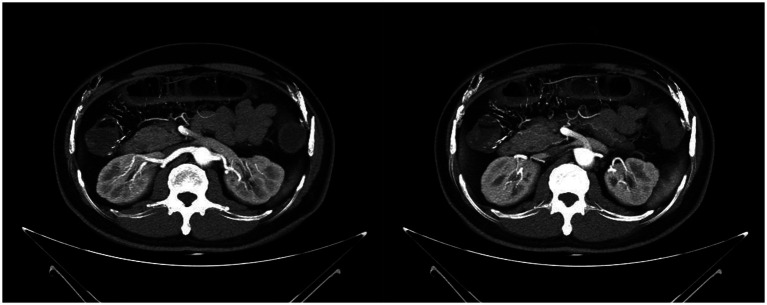
Computed tomography showed a thickened bowel wall with edema of the colonic wall in the right hemicolon, accompanied by multiple calcifications in the surrounding vasculature.

**Figure 2:**
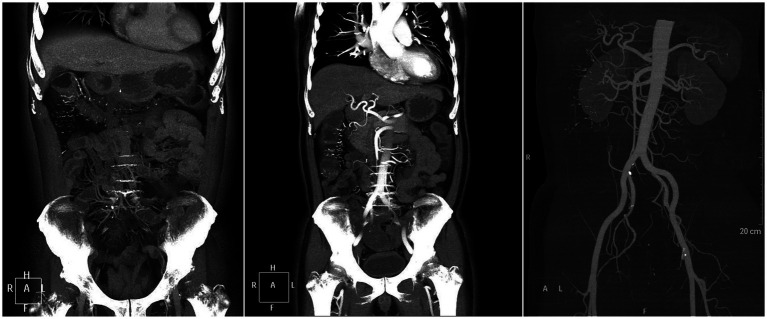
CTE demonstrates thickening of the colonic wall in the right hemicolon, with multiple short linear calcifications in the branches of the mesenteric veins.

CT demonstrated an increased number of mesenteric vein branches surrounding the ascending colon, transverse colon, and left small intestine (Figure 1). Multiple short, linear calcifications were observed in the mesenteric veins around the ascending and transverse colon (Figure 1). CTE demonstrates thickening of the colonic wall in the right hemicolon, with multiple short linear calcifications in the branches of the mesenteric veins (Figure 2).

**Figure 3:**
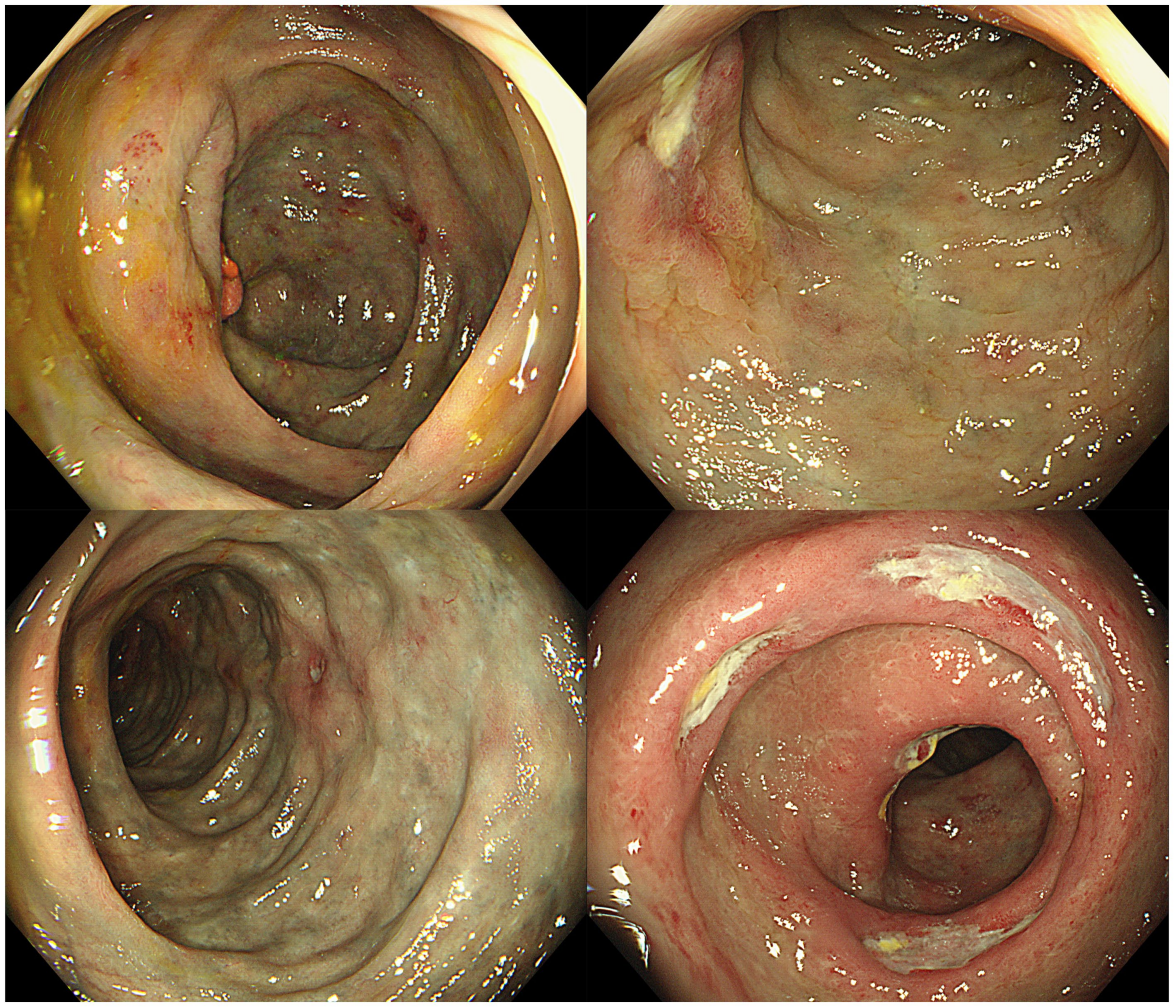
Ischemic changes with multiple ulcers in colonic mucosa.

Colonoscopy revealed that the mucosa of the entire colon and rectum was congested, edematous, and had a bluish-purple discoloration, accompanied by multiple ulcers. The surface of the ulcers was covered with a white necrotic tissue (Figure 3).

**Figure 4:**
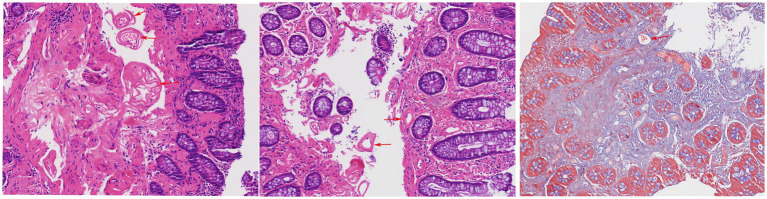
Thickening of blood vessel wall and hyalinization (indicated by red arrows). Masson’s trichrome stain demonstrates perivascular fibrosis (indicated by red arrows).

Pathological results indicated fibrous tissue proliferation in the mucosal lamina propria, with some blood vessel walls showing thickening and hyaline degeneration under microscopy. A small infiltration of lymphocytes and plasma cells was observed in the stroma. The crypt glands varied in size, but no structural abnormalities were noted. Immunohistochemistry (IHC) results were: Actin(−), Desmin(−), SMA(−). Special staining showed: Masson(+), Congo Red(−) (Figure 4).

**Figure 5:**
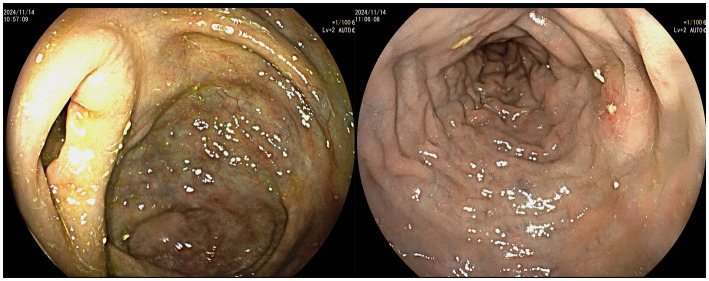
Significant improvement in colonic mucosal lesions compared to previous findings.

A final diagnosis of IMP was reached by integrating clinical characteristics with results from CT scans, colonoscopy, and histopathological analysis. The treatment plan stopped the patient’s use of traditional Chinese medicine and included mesalazine for intestinal inflammation, probiotics for gut health, aspirin for antiplatelet therapy, and atorvastatin for lowering lipids. The patient was discharged once diarrhea subsided. A follow-up colonoscopy 3 months later showed improved colorectal mucosal changes (Figure 5).

## Discussion

IMP is a rare form of chronic ischemic colitis with an unclear etiology. This disease primarily manifests as sclerosis of the mesenteric veins and their branches, leading to fibrosis, hyalinization, calcification, and thickening of the colonic wall (17). The pathogenesis of IMP may involve chronic injury to venous smooth muscle and endothelium, leading to progressive fibrosis and calcification of the vessel walls, and even gradual venous occlusion. Impaired venous return causes chronic ischemia of the intestinal wall, which in turn can induce dense fibrous connective tissue proliferation, resulting in intestinal lumen narrowing and bowel obstruction (18, 19). Radiologically, IMP typically presents with calcification of the colonic wall, especially in the right colon, a feature more commonly observed in Asian populations (20). The proposed hypothesis is that ingested toxins and biochemicals are predominantly absorbed from the right colon. Chronic stasis within the lumen leads to cephalad migration, and along with increased intraluminal pressure, causes prolonged venous return reduction, resulting in impaired venous drainage and hemorrhagic infarction of the affected colonic wall (21). Few cases have documented the pathology extending to involve the transverse colon or even the entire colon. One confirmed case involved only the left colon, with the right colon remaining unaffected (21). Although the exact etiology of IMP remains unknown, studies have suggested that prolonged use of herbal medicine, particularly those containing geniposide (22), licorice (glycyrrhizin), Scutellaria baicalensis (baicalin), Poria cocos, and alcohol may be associated with its development (17, 20, 23, 24). The consumption of herbal medicine with *sanshishi* as an ingredient has shown a strong correlation with IMP (25).

The current pathological mechanism primarily revolves around the toxic metabolite hypothesis: After being absorbed into the mesenteric veins, geniposide can induce intimal hyperplasia, thickening, and fibrosis of the venous walls, leading to the obstruction of the venous lumen and impaired intestinal venous return. This results in intestinal wall thickening and edema. Geniposide can also directly damage the intestinal wall, causing ulcer formation and fibrosis of the lamina propria. Moreover, when interacting with amino acids and/or proteins in the intestinal wall, it transforms into a dark blue pigment, which is likely responsible for the dark blue or purple discoloration of the mucosa observed during colonoscopy.

In this case, the patient took various traditional Chinese medicines orally during the period of chronic prostatitis, most of which contained geniposide. Additionally, the patient consumed these medications in doses exceeding the recommended levels for an extended period. Clinically, the symptoms of IMP are varied and non-specific, primarily including abdominal pain, bloating, and diarrhea (13). The primary clinical manifestation in this case was diarrhea, which is consistent with this condition. In the early stages, some patients might not have symptoms, but those with advanced disease could develop intestinal obstructions and even perforations (23, 26). The diagnosis of IMP primarily relies on abdominal CT and colonoscopy. Abdominal CT is crucial in identifying the characteristic tortuous thread-like calcifications in the mesenteric veins, which are indicative of IMP. These calcifications are typically seen in the right hemicolon and can extend to other parts of the colon (1, 27).

CT findings primarily include extensive punctate and linear calcifications in the areas traversed by the mesenteric veins and their branches, with the linear calcifications perpendicular to the intestinal wall. Mesenteric CTV (CT venography) more clearly demonstrates the relationship between the calcifications and the mesenteric veins. Additionally, the involved intestinal wall shows swelling and thickening, with mild uniform enhancement on contrast-enhanced scans. Some researchers have found that patients without a history of herbal medicine use exhibit slightly different features, with calcifications confined to the submucosal veins of the colonic wall rather than the mesenteric veins (12). In this case, the patient only had calcifications in the branches of the mesenteric veins, without involvement of the main trunk, which is consistent with the literature. Studies have also indicated that the extent of mesenteric venous calcification is related to the severity of the disease (6). Abdominal CT for this patient revealed diffuse thickening and edema of the entire colon and rectal walls, with visible stratification changes. Multiple calcifications were observed in the mesenteric venous routes around the ascending and transverse colon. Abdominal CTV showed increased branches of mesenteric veins around the ascending colon, transverse colon, and left small intestine, with multiple short linear calcifications seen in the mesenteric veins around the ascending and transverse colon. There were no abnormalities in the superior mesenteric vein, splenic vein, portal vein, or inferior vena cava. CT indicated multiple calcifications in the branches of the superior mesenteric vein around the ascending and transverse colon, while no significant calcifications were found in the branches of the inferior mesenteric vein. However, considering the overall edema of the colon, it is believed that the branches of the inferior mesenteric vein also had varying degrees of wall thickening and lumen narrowing, but without calcification.

The endoscopic features of IMP are characteristic and can assist in diagnosing the condition when biopsy results are inconclusive. On colonoscopy, the colonic mucosa affected by IMP displays a distinct dark purple color. Additional colonoscopic observations consist of erythema, colonic lumen narrowing, mucosal swelling, erosion, ulceration and increased rigidity of the colonic wall (26). Nonetheless, some patients may have normal colonoscopy results (28). In this case, the patient’s colonoscopy revealed that the mucosa of the entire colon and rectum was congested, edematous, and had a bluish-purple discoloration. Such cases involving the entire colon and rectum are rarer and are considered to be related to the patient’s prolonged and high-dose consumption of herbal medicines. Additionally, the patient has experienced diarrhea for many years without undergoing colonoscopic examinations or clinical treatments, leading to extensive involvement of the disease.

Due to the limited understanding of IMP, it is often challenging to distinguish it from inflammatory bowel disease and other non-neoplastic intestinal diseases in the early stages (17). Currently, there is no standard treatment regimen for IMP, with most medical centers opting for conservative management. However, for patients with recurrent symptoms who do not respond well to conservative treatment, surgical resection may be necessary (13). In some cases, postoperative pathological results confirm the diagnosis of IMP, and patients typically recover well without recurrence of IMP (13). By summarizing and analyzing the clinical and pathological features of IMP, clinicians and pathologists can improve their recognition of the disease and reduce misdiagnosis (17).

## Data Availability

The raw data supporting the conclusions of this article will be made available by the authors, without undue reservation.
